# Supplementary material for: Simple Universal Whole-Organ Resin-Embedding Protocol for Display of Anatomical Structures
Source: Biomedicines. 2023 May 12;11(5):1433. doi: 10.3390/biomedicines11051433 (PMC10216347; doi:10.3390/biomedicines11051433)
Supplement: Supplementary file 1 [file biomedicines-11-01433-s001.zip › biomedicines-2352205-supplementary/biomedicines-2352205-Supplementary Materials.pdf]

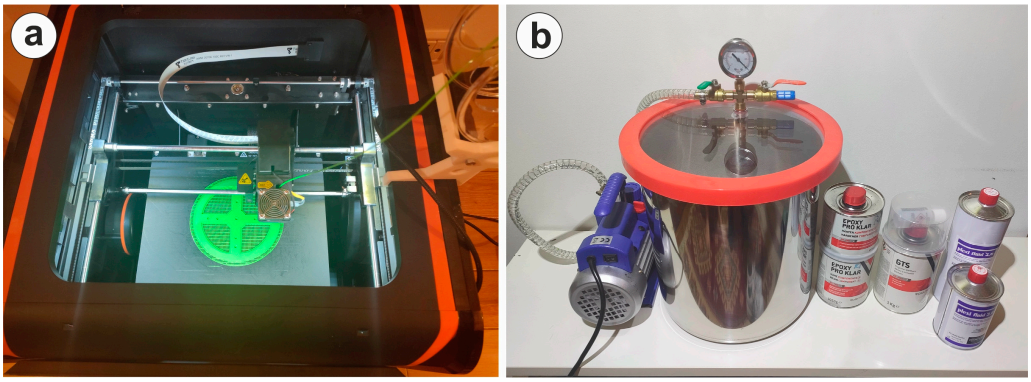

Supplementary Figure S1. (**a**) Printing the PLA support lid, and (**b**) our vacuum chamber and pump setup, according to the schematic diagram in Figure 1, together with the three resins utilized. No customization was necessary for the vacuum chamber and pump setup, everything was available as such from a local hobby store.

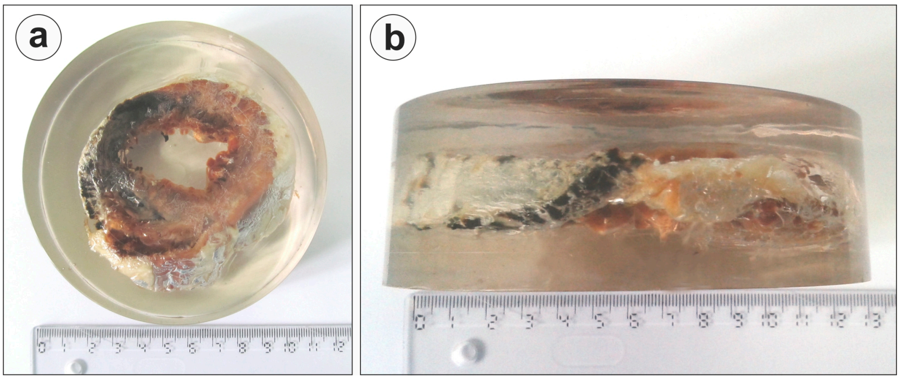

Supplementary Figure S2. Too fast polymerization leads to heating and large gas bubbles being formed around the organ, exemplified here on horizontal (a) and transversal (b) views of a heart slice (archived tissue, no glycerin processing).

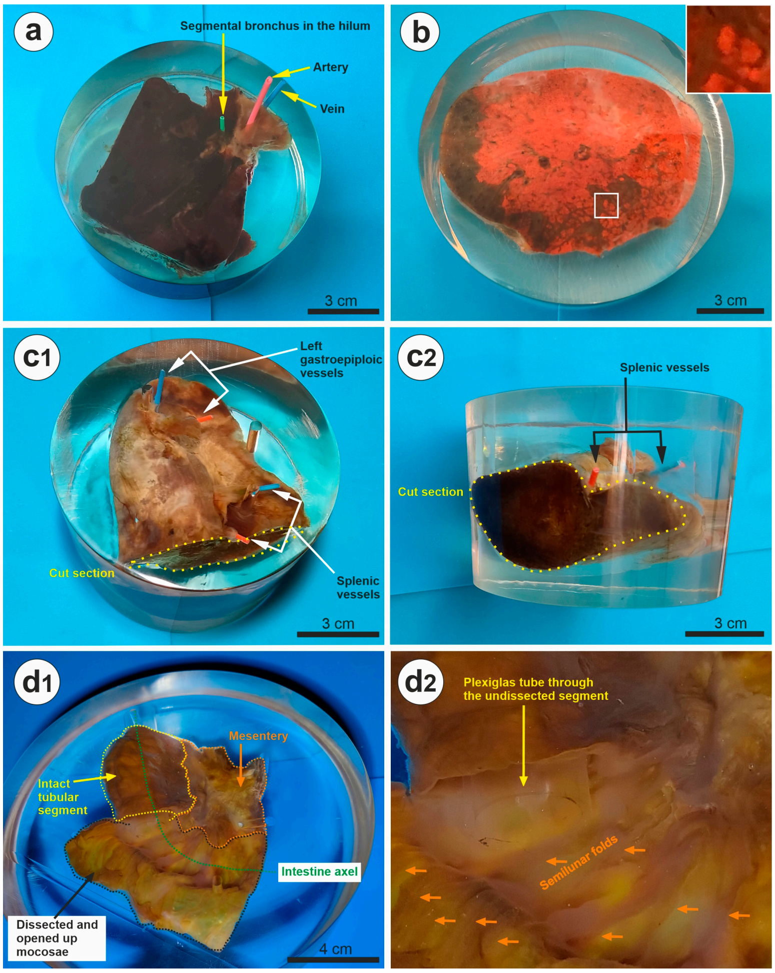

Supplementary Figure S3. Examples of other organs embedded while optimizing this protocol. Fresh 1-month fixed lung fragments processed without glycerin (**a**), or with glycerin (**b**), and injected in a segmental bronchus with a water-soluble red dye in order to show the sharp demarcation of functionally-dependent lobules (enlarged inset). (**c1**) Lower pole of a spleen (archived, glycerin processing) is visualized from above in a tall cylindrical plexiglas cast, or from a side (**c2**) illustrating its sectioned parenchyma. (**d1**) A 4 cm fragment of the transverse large intestine has been prepared with a portion of its mesentery (archived, glycerol processing), the lumen has been dissected on ½ of its length in order to expose the mucosae. (**d2**) An enlarged area of the d1 image revealing the semilunar folds of the exposed mucosae; the lumen is maintained opened through a plexiglas tube.
